# Supplementary material for: A high-resolution mRNA expression time course of embryonic development in zebrafish
Source: eLife. 2017 Nov 16;6:e30860. doi: 10.7554/eLife.30860 (PMC5690287; doi:10.7554/eLife.30860)
Supplement: Supplementary file 6. [file elife-30860-supp6.zip › biolayout-clusters-files/Cluster210-genes.html]

Cluster210


# Cluster210: Genes

| Ensembl ID | Gene Name | Chr | Start | End | Biotype |
| --- | --- | --- | --- | --- | --- |
| ENSDARG00000063233 | ENSDARG00000063233 | 17 | 9875747 | 9887229 | protein\_coding |
| ENSDARG00000035507 | ddx31 | 5 | 29114633 | 29145413 | protein\_coding |
| ENSDARG00000100666 | ep300a | 12 | 18805191 | 18839617 | protein\_coding |
| ENSDARG00000100225 | kif16ba | 1 | 50585528 | 50622737 | protein\_coding |
| ENSDARG00000000747 | lcmt2 | 9 | 27909521 | 27923332 | protein\_coding |
| ENSDARG00000071272 | ncoa6 | 23 | 2761486 | 2783712 | protein\_coding |
| ENSDARG00000086075 | ttc3 | 10 | 81893 | 108953 | protein\_coding |
